# Hydrogen Sulfide Inhibits Homocysteine-Induced Neuronal Senescence by Up-Regulation of SIRT1

**DOI:** 10.7150/ijms.38602

**Published:** 2020-01-17

**Authors:** Xuan Kang, Cheng Li, Xi Xie, Ke-Bin Zhan, San-Qiao Yang, Yi-Yun Tang, Wei Zou, Ping Zhang, Xiao-Qing Tang

**Affiliations:** 1 Institute of Neurology, the First Affiliated Hospital, University of South China, Hengyang, 42100, Hunan, P.R. China.; 2 Institute of Neuroscience, Hengyang Medical College, University of South China, Hengyang, 42100, Hunan, P.R. China.; 3 Department of Emergency, Affiliated Nanhua Hospital, University of South China, Hengyang, 421001, Hunan, P. R. China.; 4 Department of Neurology, the Second Affiliated Hospital, University of South China, Hengyang, 421001, Hunan, P.R. China.; 5 Department of Neurology, Affiliated Nanhua Hospital, University of South China, Hengyang, 421001, Hunan, P. R. China.

**Keywords:** cell senescence, homocysteine, hydrogen sulfide, SIRT1

## Abstract

Homocysteine (Hcy) accelerates neuronal senescence and induces age-related neurodegenerative diseases. Silence signal regulating factor 1 (SIRT1) prolongs lifespan and takes neuroprotective effects. We have previously demonstrated that hydrogen sulfide (H_2_S) prevents Hcy-induced apoptosis of neuronal cells and has neuroprotective effect. In the present work, we aimed to investigate whether H_2_S protects HT22 cells against Hcy-induced neuronal senescence and whether SIRT1 mediates this role of H_2_S. We found that Hcy induced cellular senescence in HT22 cells, as determined by β-galactosidase staining, expressions of P16^INK4a^, P21^CIPL^, and trypan blue Staining, which are the markers of cellular senescence. However, sodium hydrosulfide (NaHS, the donor of H_2_S) significantly reversed Hcy-induced cellular senescence. Interestingly, NaHS not only up-regulated the expression of SIRT1 in HT22 cells but also reversed Hcy-downregulated the expression of SIRT1 in HT22 cells. Furthermore, we found that pretreatment with Sirtinol (an inhibitor of SIRT1) markedly reversed the protection of NaHS against Hcy-induced HT22 cells senescence and apoptosis. Our findings illustrated that H_2_S protects HT22 cells against Hcy-induced senescence by up-regulating SIRT1.

## Introduction

Homocysteine (Hcy) is a sulfur-containing nonprotein amino acid derive from methionine metabolism 1. Hcy promotes neuronal degeneration and thus it contributes to age-related neurodegenerative diseases, such as dementia, Alzheimer's disease (AD), Parkinson's disease(PD), and stroke 2,3. The emerging evidence suggests that a potentially important contributor to aging and age-related neurodegenerative diseases is cellular senescence 4, a process that imposes permanent proliferative arrest on cells responding to various stressors 5. Therapeutic strategies that safely interfere with the detrimental effects of cellular senescence are gaining significant attention 4,6. Interestingly, accumulating evidence showed that cellular senescence is causally implicated in Hcy-generated age-related diseases 7-9. Thus, finding a new strategy to antagonize Hcy-induced neuronal senescence has important value in the prevention and treatment of age-related neurodegenerative diseases.

Hydrogen sulfide (H_2_S) is a novel gaseous molecule with an extremely unpleasant odor. In the brain, H_2_S is an important neuroprotective agent and has therapeutic potential in neurodegenerative diseases of aging, such as AD and PD 10,11. Recently, H_2_S was identifiedas a new approach to prolong lifespan 12. H_2_S inhibits the production of mitochondrial ROS 13, decreases oxidative stress 14,15, and repairs DNA damage 16 to protect cells from senescence. Previous studies revealedthat H_2_S protects endothelial cells against nicotinamide- 17 and H_2_O_2_- 18 induced cellular senescence. Our previous study indicated that H_2_S prevents Hcy-induced neurotoxicity 19,20. In this study, we explored whether ameliorating Hcy-induced neuronal senescence contributes to the protection of H_2_S against Hcy-induced neurotoxicity.

Silent mating type information regulator 2 homolog 1 (SIRT1) is a NAD+-dependent deacetylase of lysine residue of the target protein. SIRT1 extends lifespan 21 and improves cell tolerance to inhibit environmental stress 22,23. Recently, it has shown that H_2_S extends lifespan by activation of SIRT1 12. Furthermore, Our previous data demonstrated that H_2_S up-regulates the expression of SIRT1 in PC12 cells 20. Thus, we further investigated whether SIRT1 mediates the inhibitory role of H_2_S in Hcy-induced neuronal senescence.

The present studies demonstrated the ability of H_2_S to inhibit Hcy-induced senescence in HT-22 cells and identified the mediatory role of SIRT1 in the protective action of H_2_S from Hcy-induced neuronal senescence. It provides novel strategy to prevent Hcy-induced neurotoxicity via inhibition of senescence-associated neuronal aging. Thus, our research identifies SIRT-1 as a potent therapeutic target and H_2_S as a potent treatment for Hcy-related neurodegenerative diseases.

## Materials and Methods

### Materials

Sodium hydrosulfide, homocystein, sirtinol, dimethyl sulfoxide (DMSO), and propidium iodide (PI) were purchased from Sigma (St. Louis, MO, USA). Rh123 was supplied by Dojindo Molecular Technologies, Inc. (Rockvile, MD, USA). Specific monoclonal anti-SIRT1 antibody was obtained from Abcam (Hong Kong, China). Specific monoclonal antibodies of P16^INK4a^, P21^CIPL^ were purchased from OriGene Biotech Inc. (Burlingame, UK). Annexin V was bought from Nanjing Key GEN Biotech Co., Ltd. (Nanjing, China). DMEM medium, horse serum and fetal bovine serum were supplied by Gibico, BRL (Ground Island, NY, USA).

### Cell viability assay

Cell viability was detected by Cell Counting Kit-8 (CCK-8) assay. HT22 Cells of logarithmic phase growth were seeded into a 96-well plate at a density of 1 × 10^4^ cells/well. The cells were treated with 2.5, 5.0, 10.0 mM Hcy, or/and 100, 200, 400 μmol/L NaHS and routinely incubated for 24 hours, 48 hours, and 72 hours. After treatment, viable cells were stained with 5μL of CCK-8 solution (CCK-8, Sigma, USA) and incubated at 37°C for 4 h. Absorbance was measured at 450 nm by a microplate reader (ELX-800, BIO-TEK, USA). The experiment was performed three times.

### β-galactosidase (SA-β-gal) staining assay

Senescent cells were detected by the SA-β-gal staining. HT22 Cells were seeded into a 6-well plate at a density of 1 × 10^4^ cells/well. After incubated with 2ml 10% Dulbecco's Modified Eagle Medium (DMEM, Gibco, USA) for 24h, HT22 cells were treated with various concentrations of 2.5, 5.0, 10.0 mM Hcy, or/and 100, 200, 400 μmol/L NaHS for 48 h. The plates were stained daily using Senescence β-Galactosidase Staining Kit (Cell Signaling Technology, Danvers, MA, 9860) following the manufacturer's recommendations. All wells were counted in five randomized fields.

### Trypan blue Staining

The cells were grew at concentration of 1 × 10^4^ cells/ml in 24 well-plates to 60-70% confluence by incubation for 24 h, then treated with 2.5mM, 5.0mM, 10mM Hcy or and 100, 200, 400 μmol/L NaHS for 24 h, 48 h, 72 h. Floating and adhering cells were collected, washed once with PBS (pH 7.4), centrifuged, suspended in 10% DMEM and stained with 0.4% trypan blue (sigma, St. Louis, MO, USA) at room temperature. About 10 μl of sample was loaded on hemocytometer chamber and numbers of blue cells and non-blue cells were counted under a light microscope (Mike audi, China). The results were expressed as percentage of the control.

### Western blotting

Cells of logarithmic phase growth were plated in 50ml culture bottle. After incubated with DMEM for 24h, the cells were treated with Hcy or/ and NaHS at different concentrations for 48 h. Then the treated cells were resuspended in 100 uM cell lysis buffers and PMSF incubation on ice for 30 min. The supernatant was collected after centrifuged at 12,000 rpm for 10 min at 4˚C. The protein concentrations were detected by BCA method (Solarbio, Beijing, China). Equal amounts of total protein extracts were electrophoresed through 10% or 12% SDS-PAGE gel, then transferred to polyvinylidene difluoride membranes (Solarbio, China). After blocked with TBST (50 mM Tris-HCl, pH 7.4, 0.15 M NaCl, 0.1% Tween-20) containing 5% BSA (Sigma, USA) for 2 h, the membranes were incubated with primary antibodies including monoclonal antibody for P16^INK4a^, P21^CIPL^, and SIRT1(dilution, 1:1,000) at 4˚C overnight. After washed with TBST for 5 min 5 times, the membranes were incubated with secondary antibodies including Goat anti-rabbit (Proteintech, USA) (dilution, 1: 5,000) at room temperature for 2 h. After washed for three times with TBST for 3 times, the membranes were visualized with Western Blotting Chemiluminescence Reagent (Solarbio, Beijing, China), followed by apposition of the membranes with autoradiographic films (Kodak, China). The expression of β-actin for each sample was used as a control.

### Flow cytometry analysis

HT22 cells of the log phase were grew in 6-well plates and treated with Hcy or NaHS for 48h. Cells were stained 50 μg/ml propidium iodide (PI) (Roche, Mannheim, Germany) and 10 μg/ml RNase A (Sigma, St. Louis, MO, USA). Percentages of cells existing within the various phases (G0/G1, S, G2/M) of the cell cycle were calculated by the mean fluorescence intensity.

### Statistical analysis

Experiments were repeated at least three times. Values were expressed as mean ± standard error of the mean (S.E.M.). All data were analyzed by SPSS version 21.0. Data were evaluated for statistical significance with using one-way analysis of variance (ANOVA) followed by LSD post hoc tests. *P* < 0.05 was considered to indicate a statistically significant difference.

## Results

### Hcy induced the cellular senescence in HT22 cells

**Figure 1:**
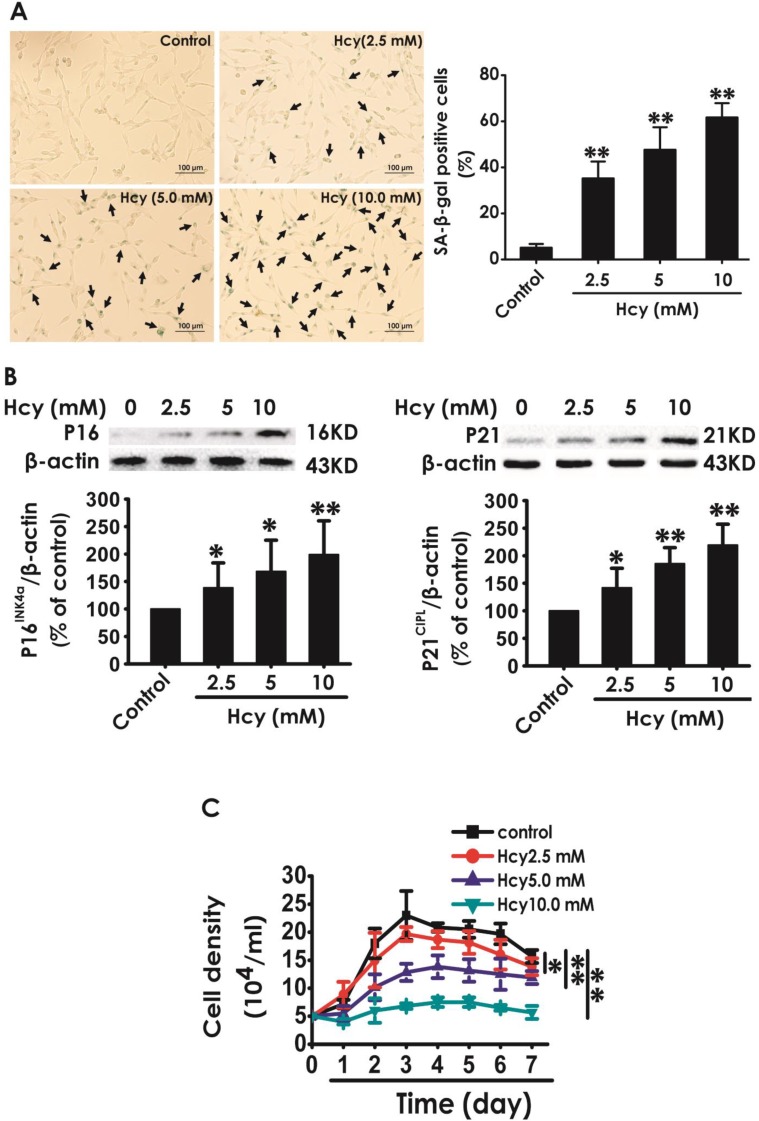
** Effect of Hcy on the cellular senescence in HT22 cells**. A, HT22 cells were stained with SA-β-gal and the SA-β-gal positive cell was quantitatively analyzed (magnification: × 10; black arrows point the SA-β-gal staining positive cells). B, the expressions of P16^INK4a^ and P21^CIPL^ in HT22 cells were measured by western blotting. C, the cell density was determined by trypan blue analysis and the growth curve for 7 d was drawn. Values are means ± SEM (n = 3). *P<0.05, **P<0.01, *vs* control group.

We first explored whether Hcy induces cellular senescence in HT22 cells. After treatment with Hcy (2.5, 5, 10 mM) for 48 h, the percentage of senescence-associated beta-galactosidase (SA-β-Gal)-positive cells was increased (Fig. 1A), the expressions of P16^INK4a^ and P21^CIPL^ were upregulated (Fig. 1B), and the cell density was decreased (Fig. 1C) in HT22 cells, which indicated that Hcy induces the cellular senescence in HT22 cells.

### H_2_S prevented Hcy-induced cellular senescence in HT22 cells

**Figure 2:**
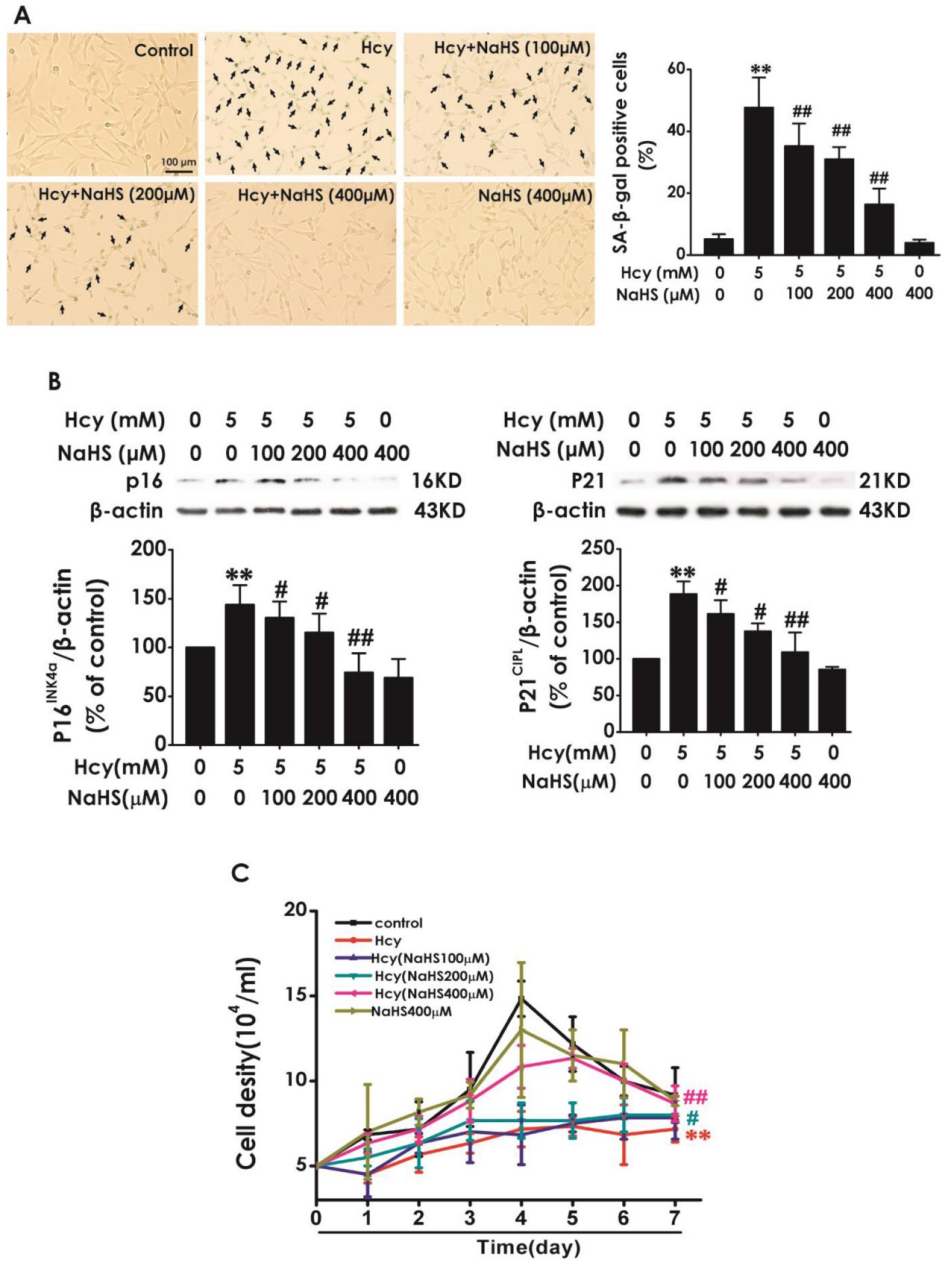
** Effect of H_2_S on Hcy-induced cellular senescence in HT22 cells.** A, HT22 cells were stained with SA-β-gal and the SA-β-gal positive cell was quantitatively analyzed (magnification: × 10; black arrows point the SA-β-gal staining positive cells). B, the expressions of P16^INK4a^ and P21^CIPL^ in HT22 cells were measured by western blotting. C, the cell density was determined by trypan blue analysis and the growth curve for 7 d was drawn. Values are means ± SEM (n = 3). **P<0.01, *vs* control group; ^#^*P*<0.05, ^#^*P*<0.01, *vs* Hcy-treated group.

Next, we explored the effect of H_2_S on Hcy-induced cellular senescence in HT22 cells. HT22 cells were pretreated with NaHS (100, 200, and 400 μM) for 30 min and then cotreated with 5 mM Hcy for 48 h. We found that pretreatment of NaHS (100, 200, or 400 mM) significantly decreased the percentage of SA-β-gal-positive cells (Fig. 2A) and the expressions of P16^INK4a^ and P21^CIPL^ (Fig. 2B), while increased the cell density (Fig. 2C) in Hcy-treated HT22 cells. These results demonstrated that H_2_S prevented Hcy-induced cellular senescence in HT22 cells.

### NaHS upregulated the expression of SIRT1 in HT22 cells

**Figure 3:**
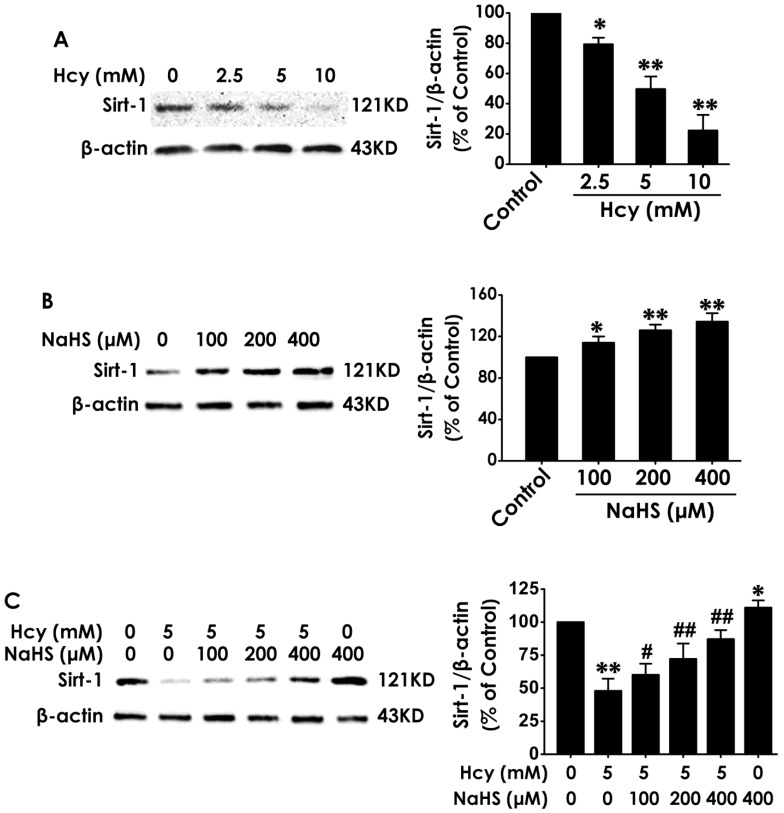
** Effects of Hcy and NaHS on the expression of SIRT1 in HT22 cells.** The expressions of SIRT1 in HT22 cells treated with 48-h Hcy (2.5, 5.0, 10.0 mM) alone (A), 48-h NaHS (100, 200, 400 μmol/L) alone (B), or 48- h cotreatment with Hcy (5.0) and NaHS (100, 200, 400 μmol/L) (C) were detected by western blotting. Values are means ± SEM (n = 3), *P<0.05, **P<0.01, *vs* control group; ^#^*P*<0.05, ^##^*P*<0.01, *vs* Hcy-treated alone group.

To explore the mediatory role of SIRT1 in the protection of H2S against Hcy-induced cellular senescence in HT22 cells, we first investigated the expression of SIRT1 in different treated HT22 cells. After the expression of SIRT1 in HT22 cells was markedly down-regulated by treatment with Hcy (2.5, 5.0, 10.0 mM) for 48 h (Fig. 3A), while was up-regulated by treatment with NaHS (100, 200, and 400 μM) alone for 48 h (Fig. 3B). Furthermore, preteatment with NaHS (100, 200, and 400 μM) restored the expression of SIRT1 in Hcy-treated HT22 cells (Fig. 3C). These results suggest that NaHS not only upregulated the expression of SIRT1 in HT22 cells but also reversed the down-regulation of SIRT1 in Hcy-treated HT22 cells.

### Sirtinol reversed the protection of NaHS against Hcy-induced cellular senescence in HT22 cells

**Figure 4:**
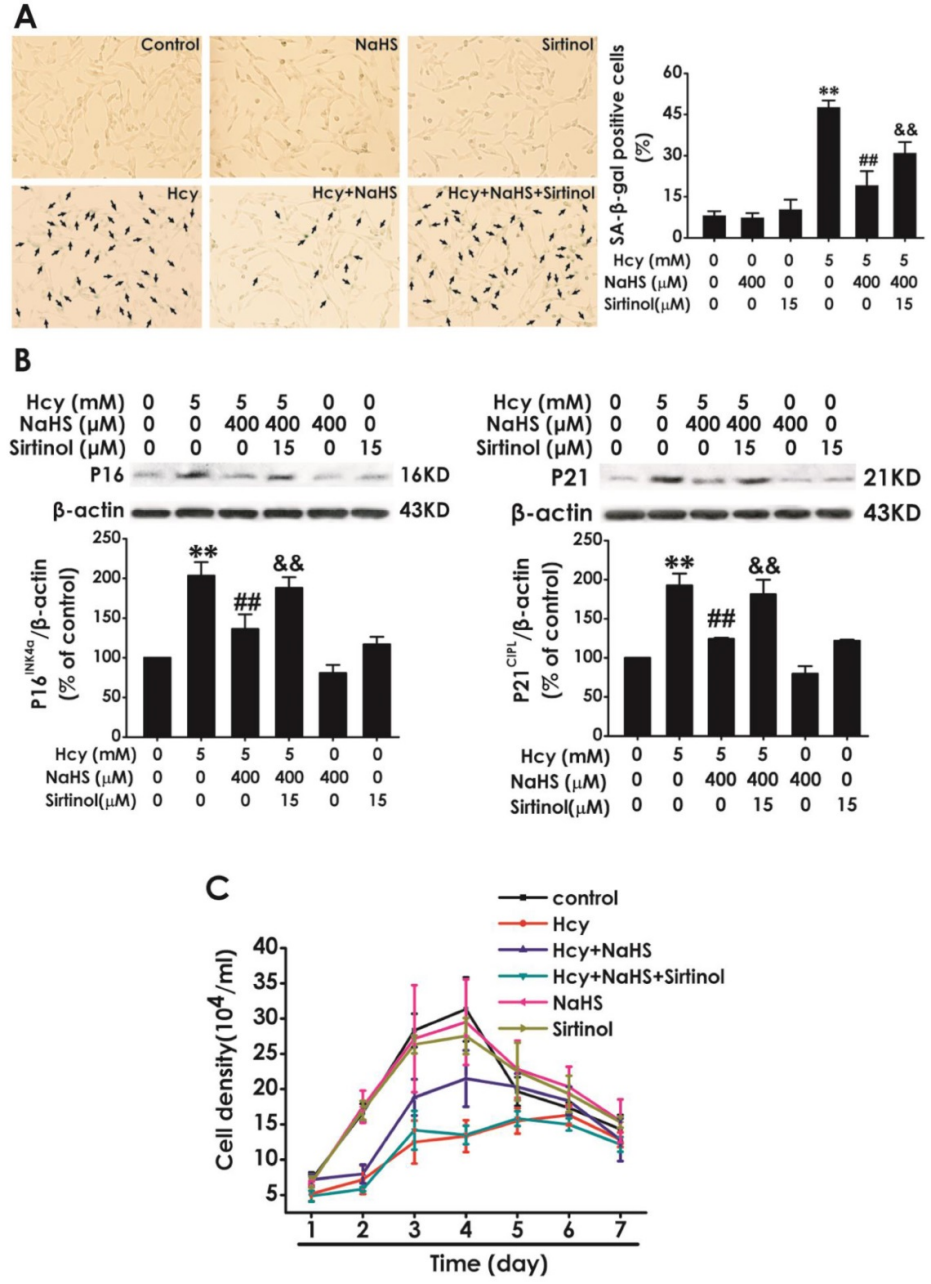
** Effect of Sirtinol on NaHS-attenuated cellular senescence in Hcy-exposed HT22 cells.** A, HT22 cells were stained with SA-β-gal and the SA-β-gal positive cell was quantitatively analyzed (magnification: × 10; black arrows point the SA-β-gal staining positive cells). B, the expressions of P16^INK4a^ and P21^CIPL^ in HT22 cells were measured by western blotting. C, the cell density was determined by trypan blue analysis and the growth curve for 7 d was drawn. Values are mean ± SEM (n = 3). **P<0.01, *vs* control group; ^##^*P*<0.01, *vs* Hcy-treated alone group; ^&^*P*<0.05 ^&&^ *P*<0.01* vs* cotreatment with Hcy and NaHS group.

To further confirm whether SIRT1 mediates the protection of NaHS against Hcy-induced cellular senescence in HT22 cells, we explored whether sirtinol, a specific inhibitor of SIRT1, reversed this protective role of NaHS. We found that pretreatment with sirtinol (15 μM, for 30 min) increased the percentage of SA-β-gal-positive cells (Fig. 4A) as well as the expressions of P16^INK4a^, P21^CIPL^ (Fig. 4B), while decreased the cell density (Fig. 4C) in HT22 cells cotreated with 5 mM Hcy and 400 μM NaHS for 48 h. These findings verified that sirtinol reverses the protection of NaHS against Hcy-induced cellular senescence in HT22 cells.

### Sirtinol reversed the protective effect of H_2_S on Hcy-indcued neurotoxicity to HT22 cells

**Figure 5:**
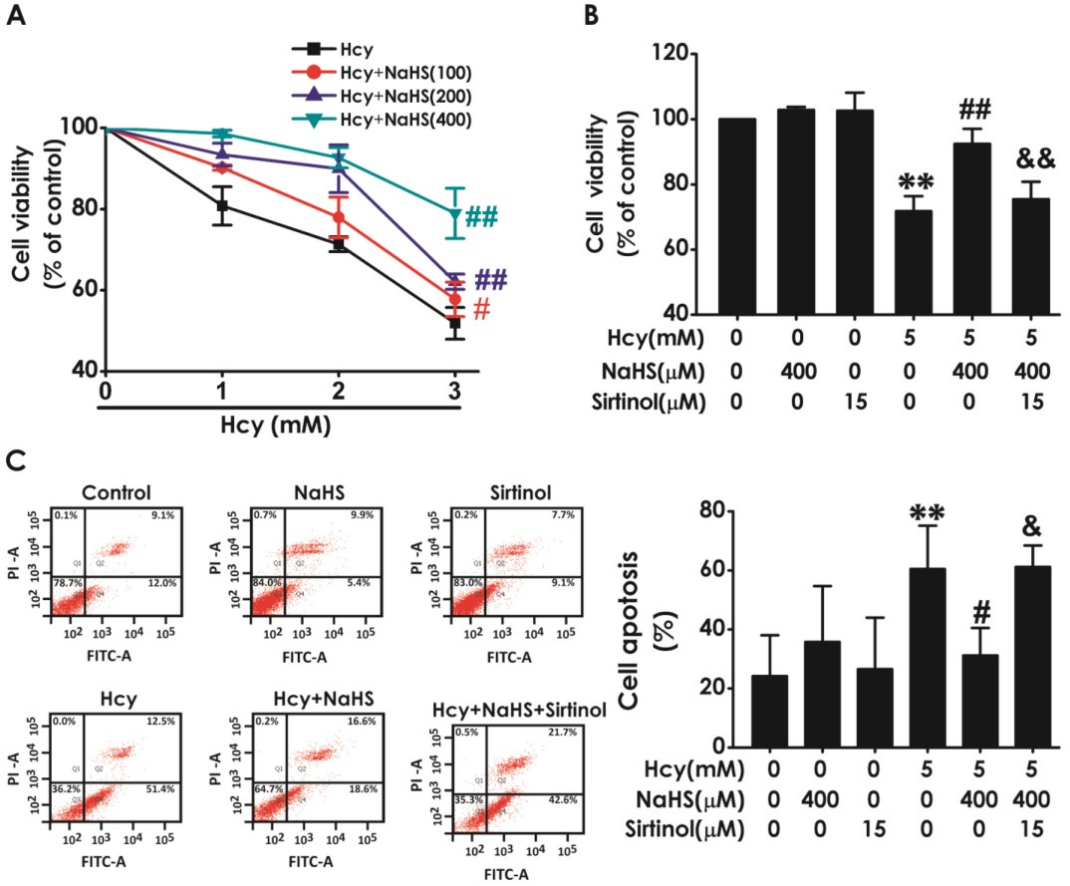
** Effect of Sirtinol on the protection of H_2_S against Hcy-induced neurotoxicity to HT22 cells**. **A**, the cells were treated with 2.5, 5.0, 10.0 mM Hcy, and 100, 200, 400 μmol/L NaHS and routinely incubated for 48 hours. **B-C**, after preincubated with Sirtinol (15 mM) for 30 min, HT22 cells were pretreated with NaHS (400 uM) for 30 min, then cotreated with Hcy (5M) for 48h. **A-B,** the cell viability was determined by CCK-8 assay. **C,** the apoptosis of HT22 cells was assessed by flow cytometry after PI and Annexin V double staining (The annexin-V2/PI2 population is made up of normal healthy cells, while annexin-V+/PI2 cells exist in early apoptotic stage, and annexin-V+/PI+ cells exist in late apoptotic stage). Values are the mean ± SEM (n = 5). *P<0.05 **P<0.01* vs* control group; ^#^*P*<0.05 ^##^*P*<0.01 *vs* Hcy-treated alone group; ^&^*P*<0.05 *vs* cotreated with NaHS and Hcy group.

We have previously demonstrated that H_2_S inhibits Hcy-induced neurotoxicity. Therefore, we next investigated whether blockage of SIRT1 reverses the protective effect of H_2_S against Hcy-induced neurotoxicity to HT22 cells. As shown in Fig. 5A, pretreatment of NaHS (100, 200, or 400 μM) significantly increased the cell viability of HT22 cells exposed to 5 mM of Hcy for 48 h, which indicated that NaHS inhibites Hcy-induced cytotoxicity to HT22 cells. However, pretreatment with sirtinol (15 μM) markedly decreased the cell viability of HT22 cells cotreated with Hcy (5 mM) and NaHS (400 μM)(Fig. 5B), which indiccated that sirtinol reverses the protection of NaHS against Hcy-induced cytotoxicity to HT22 cells. In addition, pretreatment of NaHS (400 μM, for 30 min) significantly decreased the apoptosis in HT22 cells treated with 5 mM of Hcy for 48 h, while pretreatment with sirtinol (15 μM, for 30 min) increased the apoptosis in HT22 cells cotreated with Hcy and NaHS (Fig. 5C), which indiccated that sirtinol reverses the protection of NaHS against Hcy-induced apoptosis to HT22 cells. These results demonstrated that inhibition of SIRT1 reverses the protection of H_2_S against Hcy-induced neurotoxicity in HT22 cells.

## Discussion

Our previous studies have demonstrated that H_2_S has a protective effect against Hcy-evoked neurotoxicity 20,24-26. Considering the cellular senescence is prominent in the neurotoxicity of Hcy 3,27-29, the present work was designed to explore whether the protection of H_2_S against the neurotoxicity of Hcy is associated with regulating neuronal senescence. The main findings of the present work are the following: (i) H_2_S suppressed Hcy-induced cellular senescence in HT22; (ii) H_2_S up-regulated the expression of SIRT1 in Hcy-exposed HT22 cells. (iii) Inhibition of SIRT1 reversed the protective effect of H_2_S against Hcy-induced senescence and neurotoxicity in HT22 cells. Together, we demonstrated that the protection of H_2_S against Hcy-induced neurotoxicity involves inhibition of neuronal senescence through upregulating SIRT1 signaling.

Cellular senescence, a process that imposes permanent proliferative arrest on cells in response to various stressors, has emerged as a potentially important contributor to aging and age-related disease, and it represents an attractive target for therapeutic exploitation 4. Hcy is an independent risk factor for neurological and cardiovascular disease 30,31. The level of Hcy in bodies increases with age 32. Therefore, understanding whether Hcy induces neuronal senescence is necessary for a greater understanding of the neurotoxicity of Hcy, which may be part of the mechanisms leading to age-relate neurological diseases 33,34. SA-β-Gal staining remains as the most widely used biomarker for cellular senescence 35,36. P21^CIPL^ is a cyclin-dependent kinase inhibitor, with regulating cell cycle progression at G1 and S phase 37. P16^INK4a^ plays a critical role on controlling cellular senescence 38. The present work demonstrated that the percentage of SA-β-Gal positive cells and the expressions of age-related markers P16^INK4a^ as well as P21^CIPL^ were significantly increased in Hcy-treated HT22 cells. Furthermore, we found that Hcy led to cell growth arrest. These data concluded that Hcy induces cellular senescence in HT22 cells. Therefore, modulation of neuronal senescence might represent a novel therapeutic strategy to overcome Hcy-induced neurotoxicity.

H_2_S, a well-known regulator of inflammation 39, ER stress 40 and cytotoxicity 41, is recognized as a new approach to prolong lifespan 12. H_2_S has been described to prevent endothelial cell senescence 18,42 and delays aging 43. Furthermore, we have previously found that H_2_S inhibits formaldehyde-induced cellular senescence 44 and protects against Hcy-induced neurotoxicity 25. Therefore, it is imperative to assess the effect of H_2_S on Hcy-induced cellular senescence. In the present work, we found that H_2_S increased the growth curve and decreased the percentage of SA-β-Gal positive cells as well as the expressions of P16^INK4a^ and P21^CIPL^ in Hcy-treated HT22 cells. Therefore, we for the first demonstrated that H_2_S prevents Hcy-induced cellular senescence in HT22 cells. It has been confirmed that H_2_S prevents the process of senescence in the endothelium 42, kidney 45,46, vascular 47, heart 48 and brain 43 of mice. Furthermore, increasing the content of endogenous H_2_S by proper diet extends the life span of the aged mice 49,50. These previous findings offered a reasonable explanation for the results obtained in the present study. Therefore, the regulation of neuronal senescence offers insights into the protection of H_2_S against the neurotoxicity of Hcy. In our previous work, we used PC12 cells as a cell model to explore the protection of H2S against Hcy-induced endoplasmic reticulum (ER) stress 20. This previous work focused on the protective role of H_2_S in Parkinson's disease. Thus, we used PC12 cells to explore the protection of H_2_S against Hcy-induced ER stress because PC12 cells are dopaminergic neurons and are commonly used as the cell model of PD. In the present study, the purpose is to explore the effect of H_2_S on Hcy-induced impairment in learning and memory. Learning and memory are closely related to hippocampus 51. Thus, HT22 cells were used in the present work because they are hippocampal neurons 52. Hcy may be also induced ER stress in HT22 cells. In our future, we will explore the role of ER stress in Hcy-induced neuronal senescence in HT22 cells.

The present study also investigated the possible underlying mechanism for the protective role of H_2_S in Hcy-induced senescence. Sirtuin was regarded as the lifespan-extending gene in the past years 53. SIRT1, an important member of sirtuin family, also takes an important role in delaying cellular senescence and extending longevity 29,54. Numerous studies have demonstrated that SIRT1 ameliorate neurodegenerative disease, such as AD 55, PD 56, subarachnoid hemorrhage 57, which reveal that SIRT1 delays senescence in the brain. Our previous study demonstrated that SIRT1 mediates the protective effect of H_2_S on Hcy-induced neurotoxicity 20. Therefore, we speculated whether SIRT1 mediates the protection of H_2_S against Hcy-induced senescence in HT22 cells. How might Hcy mediate the blockade of SIRT1? Previous study suggests that Hcy increases ROS generation by activating protein kinase C-β (PKCβ), which in turn improves SIRT1 degradation through a proteasome-dependent mechanism 58. In line with this finding, our present work demonstrated that Hcy downregulated the expression of SIRT1 in HT22 cells. Notably, we found that H_2_S not only increased the expressions of SIRT1 in HT22 cells, but also reversed Hcy-reduced the expressions of SIRT1 in HT22 cells. These results implied that the upregulation of SIRT1 contributed to the protective effect of H_2_S on Hcy-induced senescence. To further confirm whether the SIRT1 mediates the protection of H_2_S against Hcy-induced senescence, we explored whether the blockage of SIRT1 abolishes this protection of H_2_S. We utilized Sirtinol as the inhibitor of SIRT1, which get a broaden acceptance 59. Our results showed that inhibited SIRT1 by Sirtinol abolished the protection of H_2_S against Hcy-induced increase in the percentage of SA-β-Gal positive cells, up-regulations of P16^INK4a^ and P21^CIP1^, and the arrest of cell growth. Taken together, these results indicated that SIRT1 mediates H_2_S-exerted protection against Hcy-induced senescence in HT-22 cells. Simultaneously, the blockage of SIRT1 also eliminated the protection of H_2_S against Hcy-induced neurotoxicity, which further demonstrated that the involvement of reduced cellular senescence in the H_2_S-exerted protection against the neurotoxicity of Hcy. The possible molecular mechanism of H_2_S action on SIRT1 is unknown. A recent study has described that H_2_S increases intracellular NAD+ levels that are known to fuel SIRT1 activity 60 and that SIRT1 increases phosphorylation of extracellular signal-regulated protein kinases 1 and 2 (ERK1/2), which may be related to the regulation of anti-apoptosis and stress response 61,62. Although the precise molecular mechanisms of H2S action on SIRT1 remain to be determined, the most promising candidates are NAD+ or the pathway of PKCβ-ROS. Future works will be required to precisely define whether NAD+ and ERK1/2 implicate in the mechanisms underlying the regulatory role of H_2_S in the action of SIRT1.

## Conclusion

In this study, we demonstrated that treatment with NaHS, a donor of H_2_S, attenuated Hcy-induced cellular senescence and upregulated SIRT1 expression in Hcy-exposed HT22 cells. Furthermore, we showed the reversing role of inhibited SIRT1 in the protection of NaHS against Hcy-induced cellular senescence and neurotoxicity. These data suggested that H_2_S inhibits Hcy-induced cellular senescence by upregulation of SIRT1. Our results provide important insights into the molecular mechanism underlying H_2_S-exerted protection against Hcy-induced neurotoxicity and provide a basis for investigating H_2_S as a therapeutic approach for Hcy-related neurodegenerative disease.

## Abbreviations

Hcy: homocysteine
SIRT1: silence signal regulating factor 1
H_2_S: hydrogen sulfide
NaHS: sodium hydrosulfide
AD: Alzheimer's disease
PD: Parkinson's disease
DMSO: dimethyl sulfoxide
PI: propidium iodide
CCK-8: cell counting kit-8
DMEM: Dulbecco's Modified Eagle Medium
SA-β-Gal: senescence-associated beta-galactosidase
